# Quality of Life and Psychophysical Consequences in Individuals with Intestinal Stoma: An Observational Study

**DOI:** 10.3390/ijerph22091327

**Published:** 2025-08-26

**Authors:** Roberto Lupo, Ivan Rubbi, Annunziata Barletta, Chiara Mele, Alessia Lezzi, Carmela Triglia, Ivan Botrugno, Damiano Manca, Oscar Potì, Giuseppina Mottillo, Mirna Tondo, Giuseppe Carbotta, Giuseppe Pietro Mingolla, Claudio Marra, Maria Rosaria Tumolo, Daniele Sergi, Giorgio De Nunzio, Donato Cascio, Stefano Botti, Luana Conte, Elsa Vitale

**Affiliations:** 1 Department of Surgery, ‘San Giuseppe da Copertino’ Hospital, Local Health Authority (ASL) of Lecce, 73100 Lecce, Italy; roberto.lupo@asl.lecce.it (R.L.); ivanbot@yahoo.it (I.B.); manca.damiano@gmail.com (D.M.); oscar.poti@asl.lecce.it (O.P.); giuseppina.mottillo@asl.lecce.it (G.M.); mirna.tondo@asl.lecce.it (M.T.); giuseppe.carbotta@asl.lecce.it (G.C.); giuseppepietro.mingolla@asl.lecce.it (G.P.M.); claudio.marra@asl.lecce.it (C.M.); 2 Department of Medical and Surgical Sciences, School of Nursing, University of Bologna, 40126 Bologna, Italy; ivan.rubbi@auslromagna.it; 3 Strategic Management Staff Offices, Local Health Authority (ASL) of Lecce, 73100 Lecce, Italy; annunziata.barletta@asl.lecce.it; 4 R.S.A. “Celestino Galluccio”, 73013 Galatina, Italy; chiara.m.90@hotmail.com; 5 National Cancer Association (ANT) Italia ONLUS Foundation Lecce, 73100 Lecce, Italy; 1alessia.lezzi@gmail.com; 6 Orthopedics Ward, Ferrara Hospital, 44121 Ferrara, Italy; ce.triglia@ausl.fe.it; 7 Biological and Environmental Sciences and Technology Department, University of Salento, 73100 Lecce, Italy; mariarosaria.tumolo@unisalento.it; 8 Institute of Clinical Physiology, National Research Council, 73100 Lecce, Italy; 9 ‘Vito Fazzi’ Hospital, Local Health Authority (ASL) of Lecce, 73100 Lecce, Italy; daniele.sergi@asl.lecce.it; 10 Laboratory of Biomedical Physics and Environment, Department of Mathematics and Physics “E. De Giorgi”, University of Salento, 73100 Lecce, Italy; giorgio.denunzio@unisalento.it; 11 Advanced Data Analysis in Medicine (ADAM), Laboratory of Interdisciplinary Research Applied to Medicine (DReAM), Local Health Authority (ASL) of Lecce, 73100 Lecce, Italy; 12 Department of Physics and Chemistry “E. Segrè”, University of Palermo, 90128 Palermo, Italy; donato.cascio@unipa.it; 13 Hematology Unit, Azienda USL-IRCCS, 42122 Reggio Emilia, Italy; stefano.botti@ausl.re.it; 14 Directorate of Health and Nursing Professions, Local Health Authority (ASL) of Bari, 70100 Bari, Italy; vitaleelsa00@gmail.com

**Keywords:** intestinal stoma, quality of life, perceived stress, nursing support, enterostomal therapist

## Abstract

Background. Living with a stoma entails profound changes in a person’s life, affecting physical, psychological, and social well-being. Patients often face challenges related to body image, interpersonal relationships, and self-esteem. A stoma can impair quality of life, trigger feelings of shame and limit freedom of movement. Objectives. To assess the subjective perception of quality of life and related psychophysical consequences in individuals with an intestinal stoma. To evaluate the level of perceived support from healthcare professionals involved in the care pathway. Methods. This is a descriptive observational study conducted through the administration of an online questionnaire. The sample includes 189 adult patients with an intestinal stoma. Results. Data analysis revealed that participants aged ≥65 years and those with a permanent stoma reported higher quality of life scores compared to younger individuals or those with a temporary stoma. An inverse correlation emerged between quality of life and perceived stress (*p* < 0.001); in particular, pain and social embarrassment were strongly associated with higher levels of stress. The enterostomal therapy nurse was identified as a key figure in the care pathway (70.4%). Conclusions. The findings highlight the need for person-centered care that addresses not only clinical aspects but also emotional and relational dimensions. Enhancing the role of trained professionals, such as enterostomal therapy nurses, and promoting targeted educational interventions may contribute to improving the quality of life in patients living with a stoma.

## 1. Introduction

The concept of well-being appears to be closely intertwined with that of quality of life, particularly in the context of health-related quality of life (HRQOL), defined as “the personal judgment that encompasses the positive and negative aspects of psychological, physical, social, and spiritual well-being at a point in life when health conditions, illness, and treatments are relevant” [1]. While ostomy surgery improves survival, it frequently undermines patients’ physical, emotional, and social well-being, ultimately reducing quality of life [2,3]. Individuals living with a stoma often face varying degrees of physical, social, and psychological difficulties [3,4,5]. The impact of a stoma on well-being and quality of life is emblematic of the need to assess the outcomes of surgical procedures not only in terms of physical recovery but also with regard to the broader consequences on an individual’s life [6]. The creation of a stoma significantly affects quality of life (QoL), manifesting in physical, psychological, and social impairments [7]. Limitations in physical activity, dietary restrictions, and sexual difficulties are among the main factors affecting QoL. Although a stoma is often a life-saving intervention, patients undergo a substantial alteration of their body image. Adapting to the new condition requires the acquisition of specific technical skills for self-management, as well as a complex personal process of adjustment involving physical, emotional, relational, and spiritual dimensions. In this context, feelings such as anxiety, shame, anger, isolation, depression, stigma, and the need to accept disability emerge as key challenges that influence the psychosocial adjustment of patients [8]. It is therefore crucial to assess quality of life in these patients in order to evaluate the effectiveness of different therapeutic procedures and their overall impact—not only in clinical terms, but also on the existential experience of the individual [1,5,9].

According to the United Ostomy Associations of America (UOAA), approximately one million people in the United States live with a stoma, and around 100,000 new procedures are performed each year [10]. In Italy, the situation is more difficult to quantify due to the lack of a national registry. It is currently estimated that between 70,000 and 100,000 people live with a stoma, with around 15,000 new procedures performed annually. These figures are derived from multiple sources, including data from associations such as FAIS (Federazione Associazioni Incontinenti e Stomizzati), and from the Italian Cancer Registry (AIRTUM), which reported approximately 50,500 new cases of colorectal cancer in 2023 [11], one of the primary causes of stoma creation. The progressive increase in life expectancy, the rising prevalence of chronic diseases—including inflammatory bowel diseases—and the decline in mortality have significantly contributed to the exponential rise in these numbers.

Beyond the growing numbers, stoma patients often experience a marked deterioration in their quality of life. The literature highlights two major issues in this population: depression [12] and sexual dysfunction [13]. Pre- and post-operative education for patients and their families is essential to improve the quality of life of individuals with a stoma [5].

This study aimed to investigate patients’ perception of their quality of life and the level of perceived stress, exploring their lived experiences and evaluating the effectiveness of the care pathway. Particular attention was given to the role and adequacy of healthcare professionals involved in the care process, with the goal of identifying potential areas for improvement in the organization and delivery of care, in light of a quality of life increasingly shaped by the presence of a stoma. The research seeks to shed light on potential physical and psychological issues encountered during the rehabilitation journey and to propose improvements in care pathways and multidisciplinary management, areas that remain underexplored in the literature, particularly within the Italian context.

The objectives of this study were to assess the subjective perception of quality of life and the related psychophysical consequences in individuals living with an intestinal stoma and to evaluate the level of perceived support provided by healthcare professionals involved in their care.

## 2. Materials and Methods

### 2.1. Study Design

A cross-sectional online survey was conducted between October 2024 and April 2025.

### 2.2. Participants

The study included adult participants (>18 years) with an intestinal stoma (colostomy or ileostomy) who provided informed consent. Patients with urinary stomas, as well as those who did not consent to participate in the study or to the publication of data, were excluded. The participants in the study were able to complete the questionnaire only once, as the survey instrument was designed to be non-repeatable in order to ensure the integrity and reliability of the collected data. In addition, we screened records for potential duplicates (near-identical timestamps and sociodemographic patterns). No duplicate entries were identified. No incomplete or missing data were observed, since the completion process required a mandatory response to each item of the administered questionnaires. Participation was voluntary and without any form of financial compensation or incentive, in full compliance with ethical principles.

### 2.3. Recruitment

To carry out the study, the participation of the association “A.I.STOM.—Italian Ostomy Association” was requested. After approving the project, the association shared the survey link with its network of members, who were able to participate anonymously using devices such as smartphones, PCs, or tablets. In addition, the study involved the collaboration of several other associations, including “APIS O.d.V.—Padua Association of Incontinent and Ostomized Individuals,” “Associazione Sarda Stomizzati O.d.V., affiliated with FAIS,” and “A.L.S.I.—Lombardy Association of Ostomized and Incontinent Individuals ETS—O.d.V.” The survey link was also shared on the Facebook groups of the following pages: “Stomia S.O.S,” “Malattia di Chron—RCU…MICI,” “Stomizzati incontinenti Veneto,” “Stomia Storia Mia,” “Associazione Sunflower Project MICI e Stomia,” “Stomizzati Salerno associato FAIS,” “Stomia & Allegria—essere speranza piuttosto che avere speranza,” “I Ragazzi Della Stomia,” and “Movimento Stomizzati Bergamo”.

### 2.4. Data Collection and Instruments

A survey instrument structured in two sections was used. The first section collected sociodemographic data (age, geographic area, marital status, education level, and employment status). The second section explored various aspects, including the type of stoma and the underlying causes leading to its creation. It also included the administration of two scientifically validated questionnaires: the Short Form-12 (SF-12), Italian version—a tool originally developed in the United States to measure quality of life [14] and later translated and culturally adapted for use in Italy (IQOLA Project). This questionnaire consists of 12 items (derived from the original 36-item SF-36) and provides two scores reflecting distinct aspects of health: physical health and mental health; and the Stoma-QoL, Italian version—a questionnaire designed to assess quality of life in people with a stoma, composed of 20 questions addressing various aspects of daily life. Additionally, the Perceived Stress Scale (PSS) [15] translated into Italian by Andrea Fossati from the Vita-Salute San Raffaele University of Milan, was used to measure perceived stress. Finally, open-ended questions were included to gather suggestions for improving the care pathway for ostomized patients and to offer participants a free space to personally and directly express the meaning of living with a stoma. Qualitative analysis of open-ended responses was performed. Free-text answers were analyzed using a rapid, inductive content analysis. Two researchers independently coded responses, met to reconcile discrepancies, and generated themes by consensus.

### 2.5. Data Analysis

The analysis was conducted using Jamovi version 2.3.18 and Microsoft Office Excel. Sample characteristics were analyzed through a range of statistical measures, including frequencies, percentages, and measures of central tendency such as the mean and median, along with measures of dispersion including the standard deviation (SD) and range. Statistical significance was assessed using various methods: ANOVA (Analysis of Variance) and the *t*-test were applied to interval variables, while the chi-square test was used for nominal variables. Pearson’s correlation coefficient was employed to examine relationships between variables. A *p*-value of < 0.05 was considered statistically significant, with a 95% confidence interval. The reliability of each test was evaluated using Cronbach’s alpha to assess internal consistency. Prior to parametric testing, we assessed distributional assumptions: normality by Shapiro–Wilk and Q-Q plots, and homogeneity of variances by Levene’s test. Where assumptions were violated, we ran robust alternatives (Welch’s t/ANOVA) and non-parametric sensitivity analyses (Mann–Whitney U/Kruskal–Wallis). Results were concordant across parametric and non-parametric analyses.

### 2.6. Ethical Considerations

This study complied with all ethical and legal regulations regarding privacy and the protection of personal data. Participants were clearly informed about the purpose and implications of the study, and informed consent was obtained prior to voluntary participation. The study was approved by the Ethics Committee (Protocol No. 1084, dated 17 December 2024).

### 2.7. Internal Reliability of the Instruments Used

To ensure the methodological robustness of the study, the internal reliability and appropriateness of the instruments with respect to the analyzed sample were assessed. For this purpose, Cronbach’s alpha and the Kaiser–Meyer–Olkin (KMO) index were calculated. The results demonstrated good validity and reliability of the administered questionnaires (Table 1). Specifically, the Stoma QoL questionnaire showed excellent internal consistency (α = 0.941) and very high sampling adequacy (KMO = 0.919), confirming the strong reliability of the tool in measuring quality of life in ostomized patients. The questionnaire assessing the importance attributed to professional roles also achieved satisfactory values, with a Cronbach’s alpha of 0.826, indicating good internal consistency, and a KMO of 0.807, suggesting adequate sample representativeness. Finally, the Perceived Stress Scale showed a Cronbach’s alpha of 0.715, which denotes acceptable internal consistency, while the KMO value of 0.863 confirms good sampling adequacy. In conclusion, all three instruments were found to be valid and reliable, with values that meet the standards commonly accepted in the literature. Therefore, they may be considered robust tools not only for the current study but also for future research.

## 3. Results

This cross-sectional survey was conceived as an exploratory study. A sensitivity power analysis shows that with *N* = 189 (permanent stoma *n* = 124; temporary stoma *n* = 65), two-tailed α = 0.05, and 80% power, the study could detect between-group standardized mean differences of Cohen’s d ≈ 0.43 and correlations of r ≈ 0.20. Therefore, the sample was adequate to identify effects of at least moderate magnitude. The analyzed sample consisted of 189 individuals with an intestinal stoma. Of these, 58.2% were women (*n* = 110). The most represented area in the study was Northern Italy, accounting for 39.7% of participants (*n* = 75), followed by the South and Islands with 33.9% (*n* = 64), and Central Italy with 26.5% (*n* = 50). The most represented age group was between 45 and 65 years, comprising 47.1% of the sample (*n* = 89), followed by individuals over 65 years old at 31.7% (*n* = 60), and the younger group aged 18 to 45 years at 21.2% (*n* = 40).

A majority of participants (64.0%, *n* = 121) were married, while 17.5% (*n* = 33) were single. Smaller proportions were separated, divorced, or widowed. Regarding educational level, 53.4% (*n* = 101) had completed high school, 20.1% (*n* = 38) held a university degree, 19.0% (*n* = 36) had completed lower secondary school, 4.2% (*n* = 8) had a postgraduate education, and only 3.2% (*n* = 6) had no formal education.

In terms of employment status, 35.4% (*n* = 67) were retired, 22.2% (*n* = 42) reported having other types of occupations, 11.6% (*n* = 22) were public sector employees, and 11.1% (*n* = 21) were factory workers. Unemployed individuals accounted for 10.1% (*n* = 19), while 9.0% (*n* = 17) were self-employed. Only 0.5% (*n* = 1) were students. Sociodemographic characteristics of the population are reported in Table 2. 

Participants were asked to indicate the type of stoma they had and why it was necessary. A total of 65.6% (*n* = 124) reported having a permanent intestinal stoma, while 34.4% (*n* = 65) had a temporary stoma. The primary reason for stoma creation was cancer, accounting for 48.1% of cases (*n* = 91), followed by chronic inflammatory bowel disease in 43.9% of cases (*n* = 83). A smaller proportion (7.9%, *n* = 15) reported other medical conditions.

As shown in Table 3, participants aged ≥65 years reported significantly higher mean quality of life scores (mean = 51.5) compared to younger age groups (18–45 years: mean = 42.9), indicating a lower perceived quality of life among younger individuals. The difference across age groups was statistically significant (*p* = 0.010), confirming that age has a real impact on perceived quality of life in patients with a stoma. Regarding perceived health status, participants reported a generally good level of health (mean = 3.27, standard deviation = 0.960).

As shown in Table 4, the perception of one’s ability to perform moderately demanding physical activities was significantly associated with perceived quality of life. Patients who reported feeling physically limited had a lower Stoma-QoL score (mean = 40.4), indicating a poorer perceived quality of life. Conversely, those who did not feel limited in their daily activities reported a significantly higher quality of life (mean = 55.2).

The table highlights how limitations in work or daily activities influence quality of life in ostomized patients. Referring to Item 4—“Did you accomplish less than you would have liked?”—patients who did not perceive a reduction in their physical or functional performance had a significantly higher mean QoL score (53.6) compared to those who felt they had underperformed (41.2). In Item 5—“Did you have to limit the kind of work or other activities you do?”—those who did not report any limitations scored the highest QoL overall (57.8), while those who had to limit their activities had significantly lower scores (42.7).

Table 5 focuses on emotional well-being and how psychological status affects work performance and daily functioning in patients with a stoma. For Item 6—“Did you accomplish less than you would have liked due to emotional problems?”—participants who did not experience reduced performance due to emotional distress reported a very high QoL score (56.8). On the other hand, those who perceived a decline in performance due to emotional reasons had a substantially lower QoL (39.3). Similarly, in Item 7—“Did you have difficulty concentrating on your work or other activities?”—patients without concentration problems had high QoL scores (54.8), while those experiencing distractions, mental fatigue, or difficulty focusing reported lower QoL (40.8).

Table 6 analyzes the impact of pain on daily work and activities. Patients who did not perceive any pain-related limitations had the highest quality of life (mean = 55.8). As the impact of pain increased, QoL scores progressively decreased.

Furthermore, the relationship between perceived quality of life (Stoma-QoL) and perceived stress was examined using the Perceived Stress Scale (PSS). For each item of the Stoma-QoL, the corresponding average score on the PSS was calculated to identify potential associations between quality of life and experienced stress. The total PSS score was calculated by summing Likert-type responses (0 to 4) across 10 items, yielding a range from 0 (no stress) to 40 (maximum perceived stress). To ensure consistency in data interpretation, positively worded items (Items 4, 5, 7, 8) were reverse scored (i.e., 0 = 4; 1 = 3; 2 = 2; 3 = 1; 4 = 0), so that higher scores always reflected greater levels of stress. The percentage of perceived stress was then calculated by dividing the mean score by the maximum possible score (40) and multiplying by 100. In summary, higher total stress scores were associated with lower perceived quality of life according to the Stoma-QoL.

Table 7 highlights the social impact of living with a stoma, particularly the negative effects of isolation, embarrassment, and difficulties in social interaction, which were found to significantly reduce patients’ overall well-being. These issues are often linked to emotional problems such as reduced self-esteem, body image concerns, and fear of judgment. The integration of Stoma-QoL and PSS results revealed that individuals experiencing greater anxiety and discomfort related to their stoma (high perceived stress scores) also reported lower QoL scores.

Participants were also asked to rate the perceived importance of support from different professionals involved in the care pathway in helping them return to their “normal life” after surgery. As shown in Table 8, the most valuable resource identified during the adjustment process was inner strength, with 82.6% of participants rating it as a key factor in returning to normalcy. Conversely, peer support groups were perceived as less helpful, with only 18.5% assigning them the highest importance rating.

Overall, the results demonstrated that mean Stoma-QoL scores increased with better self-perceived health status and decreased sharply with greater physical, emotional, or social limitations. In particular, patients who reported no limitations in their daily activities (e.g., moderate physical activity, climbing stairs) or in work performance due to health problems achieved significantly higher QoL scores (mean = 55.2). Conversely, those who reported frequent or severe limitations showed markedly lower quality of life scores (*p* < 0.001).

Table 9 presents the associations between the importance attributed by ostomized patients to different professional roles and their impact on care pathways. Specifically, the roles of general nurses and enterostomal therapy nurses were compared to other figures such as physicians, psychologists, and social care assistants. Statistically significant differences were observed when comparing the perceived relevance of these professionals. The nursing role was found to be particularly important in care pathways, with the enterostomal therapy nurse being the most highly valued figure by patients.

Table 10 reported the perceived importance of professional roles in the care pathway of stoma patients.

## 4. Discussion

The primary aim of this study was to assess the health-related quality of life in patients with an intestinal stoma. When analyzing quality of life in a specific population, such as individuals with a stoma, it is essential to consider the demographic characteristics of the target group. Indeed, demographic factors such as age, marital status, place of residence, and educational level can significantly influence perceived quality of life, as widely documented in the literature [16].

The study sample included 189 participants with an intestinal stoma, mostly adult women (58.2%), with a generally high level of education and a significant proportion of retirees (35.4%). Among the variables examined, age emerged as a decisive factor. This finding contrasts with that of Neuberger L. et al. [17], who reported that in elderly populations, stoma placement negatively affects quality of life, physical ability, and functional independence. Our results suggest that younger patients may perceive the stoma as a greater obstacle, particularly in social, sexual, and professional domains, whereas older adults may demonstrate greater adaptability or different expectations.

Another relevant element is the type of stoma. Patients with a permanent stoma reported significantly higher perceived quality of life than those with a temporary one. This difference may be attributed to greater support and more stable acceptance in the former group. On the contrary, patients with temporary stomas—although intended to be reversible—tended to experience higher levels of anxiety and uncertainty, often living in a psychological state of “suspended life”, as highlighted by qualitative studies [18].

Participants in this study generally reported a neutral or positive perception of their health status, which may reflect the effectiveness of psychological adaptation and/or the support received. In daily clinical practice, the focus is often placed primarily on educating patients in stoma management, with the aim of promoting self-care, teaching them to recognize and prevent complications, and providing information regarding the prescription and distribution of medical devices, while the psychological dimension is frequently neglected or underestimated. In this study the integration of results from the Stoma Quality of Life questionnaire (Stoma-QoL) and the Short Form-12 (SF-12) provided a more comprehensive, multidimensional, and clinically meaningful assessment of quality of life in ostomized patients.

Patients experiencing persistent feelings of sadness, fatigue, or emotional interference in social activities showed notably lower QoL scores. These findings are consistent with existing literature [19,20], which underscores the strong correlation between QoL and both physical and emotional adaptability, as well as subjective perception of health status. This condition significantly hampers the resumption of daily activities, impeding both return to work and the maintenance of interpersonal relationships. As reported in the literature, difficulties in the adaptation process, along with anxiety, adversely affect stoma self-care and the performance of everyday tasks, ultimately contributing to progressive social isolation [21].

In line with the studies by Zhang Y. et al., it is essential to encourage patients to face the change with a positive attitude and to support them and their families, in order to reduce the adaptation period and improve quality of life [22].

Moreover, psychological and emotional acceptance of the stoma is strongly associated with a positive self-image. Conversely, poor acceptance is often linked to rejection and negative feelings toward one’s body and personal identity, as demonstrated by Vitale et al. As confirmed by the findings of G.M. Salomé et al. (2017) [23], the creation of a stoma is associated with body image disturbance and alterations in self-esteem, frequently accompanied by negative perceptions of one’s body and personal identity.

Pain, as discussed in Table 6, also plays a central role. Pain perceived as an obstacle to daily work significantly impairs perceived well-being and is associated with a marked decrease in quality of life, with statistically significant differences.

Stoma-QoL scores were found to be significantly correlated with scores from the Perceived Stress Scale (PSS), as shown in Table 7. As perceived stress increased, quality of life decreased (*p* < 0.001). This finding reinforces the critical role of psychological stress in stoma management, in line with literature highlighting that beyond physical consequences, stoma care requires substantial emotional and social adjustment [24].

Another objective of the study was to investigate the importance of various types of support in returning to daily life after surgery (Table 8). The results showed that inner strength was considered the most significant resource, with 68.3% of participants rating it at the maximum score (5). This finding contributes to enhancing clinical practice in the care of ostomized patients, highlighting the importance of individual resources such as resilience, self-efficacy, and a positive attitude in stoma management. In line with existing literature, self-efficacy is positively correlated with self-esteem in patients with an intestinal stoma [25]. Furthermore, this result contributes to the advancement and organization of innovative training approaches for both patients and healthcare professionals, guiding clinical practice toward the development of educational pathways aimed at strengthening patients’ self-efficacy, as also supported by previous research [26]. The importance of structured, continuous, and timely education involving patients and their families has likewise been demonstrated by a study in which the findings highlighted a significant reduction in hospital length of stay (from 7299 to 5938 days) and in readmission rates (from 12.9% to 11.2%) [27].

Family support also emerged as a key factor in adapting to the new condition, with 75.6% of participants rating family involvement as highly important in returning to everyday life. This emphasizes the central role of family networks in sustaining patients’ psychological and physical well-being. Only a minority (11.1%) considered family support unimportant, possibly reflecting situations of isolation, family conflict, or lack of a stable support system. These findings confirm the critical importance of involving and empowering the family in postoperative care and emotional support.

Friendship networks were also considered an important social resource in adapting to the new lifestyle, albeit to a slightly lesser extent than family support. This suggests that the impact of friendships may depend on subjective factors such as openness about one’s condition or the level of relational intimacy. Overall, the findings confirm that autonomy, family and organizational support, self-management, and empowerment can significantly improve patients’ quality of life [28].

Peer support groups were perceived as less helpful, suggesting a marginal role or low perceived effectiveness of these resources. This may be due to limited awareness or engagement with such services, or to subjective difficulty in opening up in group settings.

Finally, the high percentage of positive ratings confirms the central role of healthcare professionals. This highlights the importance of the therapeutic alliance and continuity of care.

Another aim of the study was to conduct a qualitative and quantitative analysis of patients’ perceptions regarding the importance of different healthcare professionals involved in the care pathway (Table 9). In particular, the roles of general nurses and specialist nurses (enterostomal therapists) were compared to those of other multidisciplinary team members: general practitioners, hospital physicians, psychologists, and healthcare assistants (HCAs) [29,30].

This comparison helped identify key touchpoints in the care process, highlight the most valued professional roles, and suggest targeted interventions to improve care quality and patient-perceived outcomes. The data revealed that the enterostomal therapist is perceived as a central figure in the care pathway: 70.4% of patients rated this role as highly important. This proportion was significantly higher than for any other category, with *p*-values < 0.001 in almost all comparisons. Trust in this figure was also supported by comparisons with general nurses, who were still valued but to a lesser extent (52.4% rated them as highly important, versus 70.4% for enterostomal therapists). This difference aligns with the literature, which recognizes the enterostomal therapist as a direct and highly competent reference for stoma-specific care [31]. Clinical assessment skills, when integrated into an educational program based on the nursing process, result in significantly greater improvements in patients’ knowledge and stoma self-care performance compared with routine education [32].

Perceptions of general practitioners and hospital physicians showed greater variability. Only 24.9% of participants rated the general practitioner as highly important, compared to 59.8% for hospital physicians. This may reflect the nature of the relationships involved: more continuous but sometimes perceived as generalist in the case of GPs, and more specialized and intervention-focused in the case of hospital physicians. Nevertheless, both medical roles were less highly rated than the enterostomal therapist, confirming that ostomized patients perceive a stronger connection and higher specific competence in the specialized nursing role [33,34].

Although the psychologist is theoretically central in supporting psychological adjustment, this figure received mostly low importance scores (55.6%). This may reflect limited access to psychological services or insufficient patient awareness of their potential benefits. A retrospective study [35] investigating the integration of psychological and nursing care found that psychological support significantly improves mood, sleep quality, psychological resilience, and self-care ability in these patients.

Lastly, the HCA, while often providing day-to-day support, was perceived as less relevant (41.8% rated them as of low importance), likely due to the less structured and continuous nature of the relationship compared to other roles.

### Study Limitations

This study is not without limitations. First and foremost, the sampling method may not guarantee a statistically representative sample of the entire Italian population. Moreover, the reliance on voluntary participation may have introduced selection bias, as individuals who chose to participate could differ in their experiences and perspectives from those who declined to take part. The study is subject to a potential recruitment bias, as the sample cannot be considered fully representative of the overall population of individuals living with a stoma. Participant enrolment through patient associations and Facebook groups may have led to an overrepresentation of individuals who are more motivated, digitally literate, and well supported socially. Consequently, the findings may not accurately reflect the condition of patients who are more socially isolated, have lower educational levels, or experience significant psychological distress. To strengthen the robustness and generalizability of the evidence, prospective studies with longitudinal follow-up are recommended, allowing for more reliable and representative data, including from underrepresented patient groups.

## 5. Conclusions

The aim of this study was to assess the quality of life of individuals living with an intestinal stoma. The findings highlighted the multiple factors that influence quality of life, even though stoma formation remains a life-saving intervention, particularly for patients affected by neoplastic or chronic diseases. The results revealed a clear correlation: as individuals’ subjective perception of their health status improves, so do their QoL (quality of life) scores, while physical and social limitations are associated with a decline in QoL.

These findings emphasize the importance of recognizing quality of life as a primary goal of nursing care, adopting a personalized, holistic, and multidimensional approach that addresses not only physical aspects but also the psychological, social, and relational dimensions of patient well-being. Future studies should focus on identifying modifiable factors that may influence physical functioning, quality of life, and independence from the stoma.

## Figures and Tables

**Table 1:** Internal reliability of the instruments used.

Instrument	α	KMO
Stoma QoL	0.941	0.919
Importance of Professional Profile	0.826	0.807
Perceived Stress Scale	0.715	0.863

**Table 2:** Sociodemographic characteristics of the sample (*n* = 189).

	*n*	%
Gender:		
Male	79	41.8%
Female	110	58.2%
Geographical origin		
North	75	39.7%
Centre	50	26.5%
South and Islands	64	33.9%
Age Group		
≥18–˂45	40	21.2%
≥45–˂65	89	47.1%
≥65	60	31.7%
Marital Status		
Single	33	17.5%
Married	121	64.0%
Divorced	16	8.5%
Separated	7	3.7%
Widowed	12	6.3 %
Education Level:		
No formal education	6	3.2%
Lower secondary school certificate	36	19.0%
High school diploma	101	53.4%
University degree	38	20.1%
Postgraduate education	8	4.2%
Occupational Status		
Unemployed	19	10.1%
Student	1	0.5%
Manual worker	21	11.1%
Public employee	22	11.6%
Freelancer	17	9.0%
Retired	67	35.4%
Other	42	22.2%

**Table 3:** Type and cause of stoma formation.

The Stoma You Received Is:		
Permanent	124	65.6%
Temporary	65	34.4%
**What was the reason for your intestinal stoma placement?**		
Tumor	91	48.1%
Intestinal disease	83	43.9%
Other	15	7.9%

**Table 4:** SF-12 quality of life analysis.

SF-12. Overall, Would You Say Your Health Is:	
N	189
Mean	3.27
Median	3
Standard Deviation	0.960
Min	1
Max	5

**Table 5:** Quality of life and physical activity.

2. SF-12-The following questions concern activities you might do during a typical day. [Moderate activities, such as moving a table, using a vacuum cleaner, bowling, or riding a bicycle]				<0.001 **
Yes, limited a lot	46	40.4	13.2	
Yes, limited a little	77	44.3	13.0	
No, not limited at all	66	55.2	12.9	
3. SF-12-The following questions concern activities you might do during a typical day. [Climbing several flights of stairs]				<0.001 **
Yes, limited a lot	33	40.1	13.2	
Yes, limited a little	64	44.5	14.1	
No, not limited at all	92	51.6	13.5	
4. SF-12-Did you accomplish less than you would have liked?				<0.001 **
No	91	53.6	12.9	
Yes	98	41.2	12.9	
5. SF-12-Did you have to limit the kind of work or other activities you do?				<0.001 **
No	56	57.8	12.2	
Yes	133	42.7	12.7	

**: statistical significant

**Table 6:** Emotional well-being and performance.

9. SF-12-During the past four weeks, how often have you felt… [calm and peaceful]?				<0.001 **
Always	14	63.8	13.8	
Almost always	61	51.9	13.5	
Often	34	46.5	11.3	
Sometimes	55	40.1	12.7	
Almost never	20	43.6	12.7	
Never	5	39.4	12.6	
10. SF-12-During the past four weeks, how often have you felt… [full of energy]?				<0.001 **
Always	12	51.2	18.4	
Almost always	45	53.9	13.9	
Often	31	47.0	13.0	
Sometimes	57	44.9	13.0	
Almost never	30	40.4	13.8	
Never	14	46.5	13.5	
11. SF-12-During the past four weeks, how often have you felt… [downhearted and depressed]?				<0.001 **
Always	11	32.3	8.67	
Almost always	28	40.0	11.38	
Often	26	44.5	15.84	
Sometimes	61	44.4	11.22	
Almost never	44	54.9	12.20	
Never	19	61.4	13.07	

**: statistical significant

**Table 7:** Pain interference.

8. SF-12-During the past four weeks, how much did pain interfere with your normal work (including both work outside the home and housework)?				<0.001 **
Not at all	69	55.8	12.0	
Very little	38	48.8	13.1	
A little	39	41.1	11.7	
Quite a lot	22	37.6	12.9	
Extremely	21	37.3	12.4	

**: statistical significant

**Table 8:** Perceived stress and quality of life.

	Perceived Stress Scale			
	N	Mean	SD	
Stoma-Qol 1				
Always	89	21.6	6.49	F = 12.4; *p* = <0.001
Sometimes	58	18.5	7.38	
Rarely	27	14.5	7.73	
Never	15	11.9	8.78	
Stoma-Qol 2				
Always	78	22.4	7.19	F = 14.0; *p* = <0.001
Sometimes	68	17.9	6.69	
Rarely	30	14.3	7.50	
Never	13	13.2	7.24	
Stoma-Qol 3				
Always	88	21.4	7.82	F = 7.45; *p* = <0.001
Sometimes	51	16.7	7.14	
Rarely	33	17.6	6.59	
Never	17	14.3	7.28	
Stoma-Qol 4				
Always	59	21.9	6.57	F = 8.90; *p* = <0.001
Sometimes	57	19.4	6.82	
Rarely	47	17.5	8.04	
Never	26	13.3	8.54	
Stoma-Qol 5				
Always	59	21.4	6.52	F = 8.83; *p* = <0.001
Sometimes	61	20.0	6.58	
Rarely	39	17.4	8.64	
Never	30	13.5	8.32	
Stoma-Qol 6				
Always	57	22.1	7.37	F = 16.6; *p* = <0.001
Sometimes	70	20.3	6.83	
Rarely	46	15.7	6.19	
Never	16	10.2	7.94	
Stoma-Qol 7				
Always	77	22.6	6.51	F = 16.5; *p* = <0.001
Sometimes	64	18.2	7.33	
Rarely	30	14.9	6.92	
Never	18	12.1	7.42	
Stoma-Qol 8				
Always	55	23.2	7.40	F = 17.3; *p* = <0.001
Sometimes	76	19.5	5.75	
Rarely	43	13.6	6.29	
Never	15	14.8	11.14	
Stoma-Qol 9				
Always	89	22.5	7.31	F = 16.7; *p* = <0.001
Sometimes	43	16.6	5.92	
Rarely	27	16.6	6.68	
Never	30	13.4	7.39	
Stoma-Qol 10				
Always	56	24.2	6.31	F = 20.5; *p* = <0.001
Sometimes	62	18.6	6.06	
Rarely	48	15.5	8.23	
Never	23	13.4	6.36	
Stoma-Qol 11				
Always	33	22.9	6.31	F = 8.12; *p* = <0.001
Sometimes	54	20.1	7.06	
Rarely	47	18.7	6.72	
Never	55	15.3	8.62	
Stoma-Qol 12				
Always	50	24.4	6.21	F = 25.0; *p* = <0.001
Sometimes	54	20.2	5.74	
Rarely	41	16.4	7.87	
Never	44	13.3	6.74	
Stoma-Qol 13				
Always	46	23.3	7.01	F = 21.0; *p* = <0.001
Sometimes	49	21.7	5.71	
Rarely	46	17.0	7.73	
Never	48	13.5	6.49	
Stoma-Qol 14				
Always	31	24.1	6.39	F = 16.7; *p* = <0.001
Sometimes	52	20.9	5.61	
Rarely	55	18.8	8.59	
Never	51	13.7	6.48	
Stoma-Qol 15				
Always	63	24.3	6.67	F = 30.3; *p* = <0.001
Sometimes	42	19.2	6.42	
Rarely	46	16.5	6.11	
Never	38	12.4	6.30	
Stoma-Qol 16				
Always	45	24.7	7.09	F = 22.0; *p* = <0.001
Sometimes	40	20.2	5.59	
Rarely	44	18.2	7.20	
Never	60	14.1	6.75	
Stoma-Qol 17				
Always	27	26.2	6.45	F = 26.3; *p* = <0.001
Sometimes	45	21.6	6.48	
Rarely	57	18.6	6.83	
Never	60	13.7	6.38	
Stoma-Qol 18				
Always	26	25.9	6.22	F = 21.8; *p* = <0.001
Sometimes	37	22.7	5.24	
Rarely	46	18.3	6.91	
Never	80	15.1	7.35	
Stoma-Qol 19				
Always	29	24.9	6.06	F = 27.5; *p* = <0.001
Sometimes	33	23.6	5.61	
Rarely	64	18.8	6.67	
Never	63	13.7	6.96	
Stoma-Qol 20				
Always	20	23.5	6.46	F = 10.2; *p* = <0.001
Sometimes	35	21.9	5.30	
Rarely	54	19.8	7.77	
Never	80	15.7	7.78	

**Table 9:** Perceived importance of support in recovery.

	*n*	%
Parental relationships		
1	12	6.3%
2	9	4.8%
3	25	13.2%
4	35	18.5%
5	108	57.1%
Friendship relationships		
1	29	15.3%
2	14	7.4%
3	28	14.8%
4	53	28.0%
5	65	34.4%
Relationships with professionals (doctors, nurses)		
1	19	10.1%
2	19	10.1%
3	37	19.6%
4	45	23.8%
5	69	36.5%
Support groups (peer support)		
1	74	39.2%
2	23	12.2%
3	32	16.9%
4	25	13.2%
5	35	18.5%
Inner strength		
1	5	2.6%
2	10	5.3%
3	18	9.5%
4	27	14.3%
5	129	68.3%

**Table 10:** Perceived importance of professional roles in the care pathway of stoma patients.

	**Enterostomal Therapist**				**Nurse**				
	**Low**	**Medium**	**High**		**Low**	**Medium**	**High**		
	***n* = 26 (13.8%)**	***n* = 30 (15.9%)**	***n* = 133 (70.4%)**		***n* = 40 (21.2%)**	***n* = 50 (26.5%)**	***n* = 99 (52.4%)**		
	**N (%)**			***p***	**N (%)**			***p***	**Total**
General Practitioner									N = 189 (100)
Low = 107 (56.6%)	18 (9.5)	20 (10.6)	69 (36.5)	0.007 **	33 (17.5)	34 (18.0)	40 (21.2)	<0.001 **	107 (56.6)
Medium = 35 (18.5%)	7 (3.7)	7 (3.7)	21 (11.1)		4 (2.1)	11 (5.8)	20 (10.6)		35 (18.5)
High = 47 (24.9%)	1 (0.5)	3 (1.6)	43 (22.8)		3 (1.6)	5 (2.6)	39 (20.6)		47 (24.9)
Hospital Physician									
Low = 37 (19.6%)	11 (5.8)	11 (5.8)	15 (7.9)	<0.001 **	22 (11.6)	11 (5.8)	4 (2.1)	<0.001 **	37 (19.6)
Medium = 39 (20.6%)	7 (3.7)	15 (7.9)	17 (9.0)		9 (4.8)	20 (10.6)	10 (5.3)		39 (20.6)
High = 113 (59.8%)	8 (4.2)	4 (2.1)	101 (53.4)		9 (4.8)	19 (10.1)	85 (45.0)		113 (59.8)
Psychologist									
Low = 105 (55.6%)	22 (11.6)	17 (9.0)	66 (34.9)	<0.001 **	32 (16.9)	29 (15.3)	44 (23.3)	<0.001 **	105 (55.6)
Medium = 27 (14.3%)	1 (0.5)	12 (6.3)	14 (7.4)		3 (1.6)	12 (6.3)	12 (6.3)		27 (14.3)
High = 57 (30.2%)	3 (1.6)	1 (0.5)	53 (28.0)		5 (2.6)	9 (4.8)	43 (22.8)		57 (30.2)
Healthcare Assistant (OSS)									
Low = 79 (41.8%)	18 (9.5)	17 (9.0)	44 (23.3)	<0.001 **	38 (20.1)	26 (13.8)	15 (7.9)	<0.001 **	79 (41.8)
Medium = 41 (21.7%)	5 (2.6)	12 (6.3)	24 (12.7)		2 (1.1)	23 (12.2)	16 (8.5)		41 (21.7)
High = 69 (36.5%)	3 (1.6)	1 (0.5)	65 (34.4)		--	1 (0.5)	68 (36.0)		69 (36.5)

**: statistical significant

## Data Availability

The original contributions presented in this study are included in the article. Further inquiries can be directed to the corresponding author.
